# DNA Damage Response is Prominent in Ovarian High-Grade Serous Carcinomas, Especially Those with Rsf-1 (HBXAP) Overexpression

**DOI:** 10.1155/2012/621685

**Published:** 2011-10-19

**Authors:** Malti Kshirsagar, Wei Jiang, Ie-Ming Shih

**Affiliations:** ^1^Department of Pathology, Johns Hopkins Medical Institutions, Baltimore, MD 21231, USA; ^2^Department of Pathology, Cleveland Clinics, Cleveland, OH 44195, USA

## Abstract

DNA damage commonly occurs in cancer cells as a result of endogenous and tumor microenvironmental stress. In this study, we applied immunohistochemistry to study the expression of phosphorylated Chk2 (pChk2), a surrogate marker of the DNA damage response, in high grade and low grade of ovarian serous carcinoma. A phospho-specific antibody specific for threonine 68 of Chk2 was used for immunohistochemistry on a total of 292 ovarian carcinoma tissues including 250 high-grade and 42 low-grade serous carcinomas. Immunostaining intensity was correlated with clinicopathological features. We found that there was a significant correlation between pChk2 immunostaining intensity and percentage of pChk2 positive cells in tumors and demonstrated that high-grade serous carcinomas expressed an elevated level of pChk2 as compared to low-grade serous carcinomas. Normal ovarian, fallopian tube, ovarian cyst, and serous borderline tumors did not show detectable pChk2 immunoreactivity. There was no significant difference in pChk2 immunoreactivity between primary and recurrent high-grade serous carcinomas. In high-grade serous carcinomas, a significant correlation (*P* < 0.0001) in expression level (both in intensity and percentage) was found between pChk2 and Rsf-1 (HBXAP), a gene involved in chromatin remodeling that is amplified in high-grade serous carcinoma. Our results suggest that the DNA damage response is common in high-grade ovarian serous carcinomas, especially those with Rsf-1 overexpression, suggesting that Rsf-1 may be associated with DNA damage response in high-grade serous carcinomas.

## 1. Introduction

Ovarian carcinomas comprise a diverse group of neoplasms that demonstrate distinct clinicopathological features and unique molecular genetic aberrations with respect to different histologic subtypes [1, 2]. Based on clinicopathological and molecular genetic features, we have previously proposed that ovarian carcinoma can be classified into two major types, type I (low-grade serous, low-grade endometrioid, clear cell, and mucinous carcinomas) and type II (mainly high-grade serous carcinomas) [3]. Given the fact that different subtypes of ovarian tumors develop along distinct molecular pathways, we asked if the DNA damage response (DDR) is different among high-grade and low-grade serous carcinomas, the prototypes of type II and type I tumor, respectively. 

It has been well established that the DDR pathway is activated by endogenous and environmental cellular stress that is associated with DNA damage and has profound effects on determining cell fate. DDR signaling has been reported to be associated with several types of human cancer including colorectal, pancreatic, and oral squamous cell carcinomas [4–6]. Given its critical role in tumor development, it has been proposed that harnessing the activity of DDR pathways may improve cancer treatment outcome after cytotoxic chemotherapy and irradiation therapy [7]. Molecularly, DDR is initiated by the rapid recruitment of several nuclear proteins involved in the repair process to the site of DNA damage to form a complex, which acts to repair DNA damage and promote cell survival. DDR is mediated by a signal transduction cascade involving the ataxia telangiectasia mutated (ATM-) check point kinase 2 (Chk2)-p53 axis [8, 9]. In this cascade, Chk2 plays a pivotal role; ATM phosphorylates Chk2 to generate pChk2, the active form of Chk2, which then activates several downstream pathways, leading to cell cycle arrest through p53, BRCA1, Cdc25A, and Cdc25C phosphatase [10, 11]. Because threonine 68 of Chk2 is phosphorylated at sites of DNA strand breaks and the specific antibody that binds pChk2 at threonine 68 is available [12], pChk2 immunoreactivity has been used in many studies as a surrogate tissue biomarker for DDR [4–6, 13, 14]. In fact, it has been reported that Chk2 with Thr 68 phosphorylation was detected in more than 50% of primary untreated lung and breast tumor specimens [15].

In this study, we address three main questions: whether there is a difference in the level of DDR between high-grade and low-grade serous carcinoma; whether recurrent high-grade serous carcinomas have an altered DDR as compared to their primaries; whether there is a significant correlation in the expression levels between pChk2 and Rsf-1 (HBXAP), a gene that is frequently upregulated in high-grade serous carcinoma and participates in generating DNA damage. The Rsf-1 gene, located at ch11q13.5, is frequently amplified, and its expression is upregulated in most high-grade serous carcinomas but not in type I tumors [16–18]. Here we applied pChk2 immunohistochemistry to assess the levels of DDR in high-grade serous carcinoma, low-grade serous carcinoma, serous borderline tumor, and serous cystadenoma. Comparing the levels of DDR in cancer cells should help understand the pathogenesis of different subtypes of ovarian serous carcinoma.

## 2. Methods

An antibody specific for threonine 68 (Thr 68) of Chk2 (Cell Signaling, Danvers, Mass) was used for immunohistochemistry on ovarian carcinoma tissues at a dilution of 1 : 200. Phosphorylation of Chk2 at Thr 68 is a prerequisite for full activation by ATM (3). The specificity of the antibody was reported in a previous study [19]. A total of 292 ovarian carcinomas were analyzed, including 250 high-grade serous carcinomas (159 primary and 91 recurrent tumors) and 42 low-grade serous carcinomas. In addition, normal ovaries and fallopian tubes from 5 patients, 10 serous cystadenomas, and 16 serous (atypical proliferative) borderline tumors were also analyzed. All the ovarian tumors tissues except 14 low-grade serous tumors were arranged in tissue microarrays in triplicate (1.5 mm core) to facilitate immunohistochemistry. pChk2 immunoreactivity was semiquantitatively scored by two pathologists using a four-tiered grading system (0 to 3+) for intensity and percentage for prevalence of positive cells. Correlations of intensity and percentage of pChk2 with clinical data including grade (Chi square), disease-free interval, and overall survival (Kaplan-Meier curves) were determined. A monoclonal anti-Rsf-1 antibody, clone 5H2/E4 (Upstate, Lake Placid, NY), was used for immunostaining in 75 high-grade ovarian serous carcinomas arranged in tissue microarrays. Rsf-1 immunoreactivity was semiquantitatively scored by two pathologists independently using a five-tier grading system (0 to 4+) as previously described [17]. A nonparametric Kruskal-Wallis test was used to determine the statistical significance of correlation between pChk2 expression (both intensity and percentage) and Rsf-1 immunostaining intensity. 

## 3. Results

**Figure 1:**
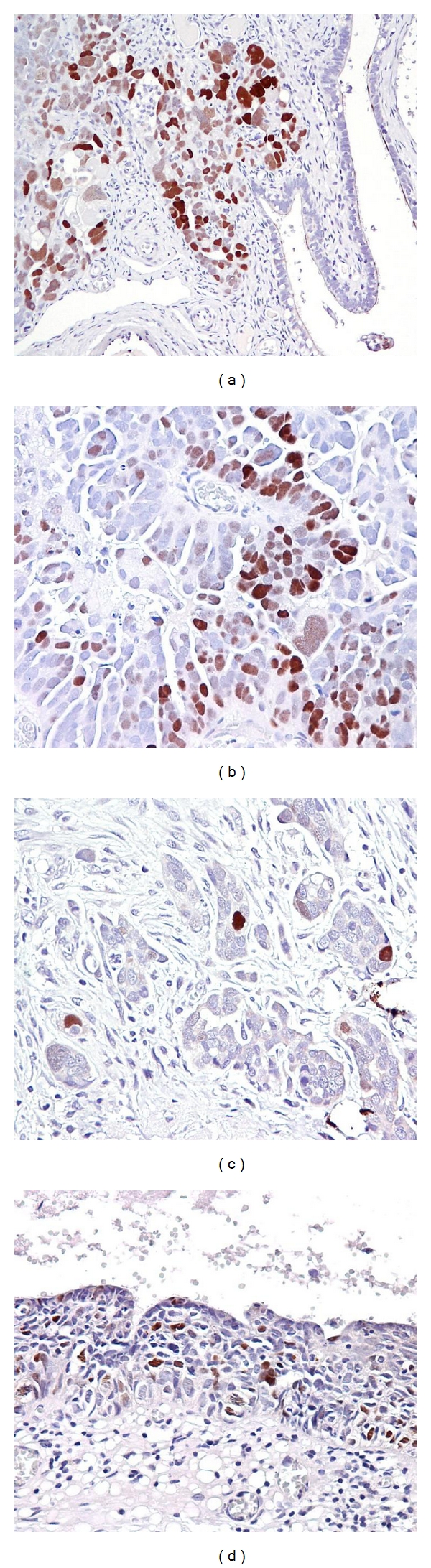
pChk2 immunoreactivity in representative high-grade serous carcinomas. (a) A high-grade serous carcinoma shows relatively diffuse positive staining for pChk2. In contrast, pChk2 immunoreactivity is undetectable in the adjacent fallopian tube epithelium. (b) At a higher magnification, pChk2 immunoreactivity is exclusively present in the nuclei of tumor cells. (c) Another high-grade serous carcinoma shows focal pChk2 staining in cancer cells. (d) A serous tubal intraepithelial carcinoma, a presumable precursor of high-grade serous carcinoma, contains pChk2 positive cells in the lesion.

**Figure 2:**
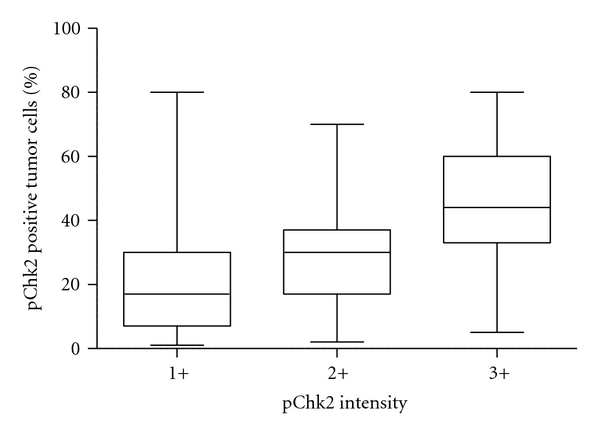
Box plot for the correlation of pChk2 immunostaining intensity and percentage of tumor cells showing pChk2 staining among 206 ovarian tumor tissues. The percentage of pChk2 positive cells correlates with intensity grade: percent pChk2 positive cells in 3+ cases > 2+ cases > 1+ cases (*P* < 0.0001, nonparametric one-way ANOVA test).

**Figure 3:**
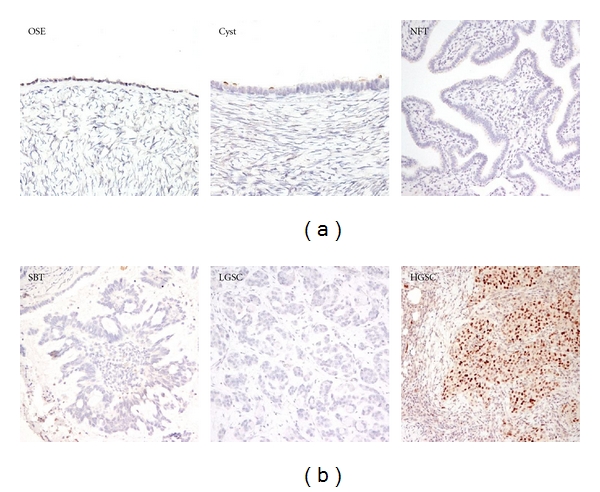
pChk2 immunoreactivity in ovarian tissues and different histologic subtypes of ovarian carcinomas. (a) pChk2 staining is undetectable in ovarian surface epithelium (OSE), cystadenoma (Cyst), normal fallopian tube (NFT). (b) serous borderline tumor (SBT) and low-grade serous carcinoma (LGSC), are negative for pChk2 staining. In contrast, a high-grade serous carcinoma (HGSC) is diffusely positive for pChk2.

pChk2 immunoreactivity was exclusively localized in the nuclei of tumor cells (Figure 1). In general, the intensity of pChk2 and the percentage of pChk2 positive cells varied among tumor samples (Figures 1(a)–1(c)) but were highly correlated. The percentage of positive cells in 3+ cases was greater than that in 2+ cases which in turn was higher than that in 1+ cases (*P* < 0.0001, nonparametric one-way ANOVA test) (Figure 2). For example, among high-grade serous carcinomas, using a percentage of 30% as an arbitrary cutoff, 77% of 3+ tumors, 33% of 2+ tumors, and 21% of 1+ tumors contained pChk2 positive cells in more than 30% of cells. The pChk2 immunoreactivity in different types of ovarian serous tumors and normal tissues was summarized in Table 1. Comparing low-grade to high-grade carcinomas, we found that 66 (26.4%) of 250 high-grade serous carcinomas, including primary and recurrent tumors, showed intense pChk2 immunoreactivity (3+) while only 3 (7.1%) of 42 low-grade serous carcinomas had immunostaining scores of 3+. If the cutoff of <2 versus ≥2 was used, 123 of 250 (49.2%) type II carcinomas and 6 (14.2%) of 42 low-grade serous carcinomas had scores ≥2. As compared to low-grade carcinomas, high-grade serous carcinomas demonstrated a statistically higher frequency of intense staining with an intensity score >2 (*P* = 0.0002, Chi-square, two-sided). In high-grade serous carcinomas, no significant difference in staining intensity or percentage was observed between recurrent and primary tumors based on different cutoffs of intensity score and percentage of pChk2 positive cells. In contrast to carcinoma tissues, ovarian surface epithelium, ovarian surface inclusion cysts, serous cystadenomas, serous borderline tumors, and tumor stromal tissues showed undetectable levels of pChk2 immunoreactivity (Figure 3). There was no significant association of pChk2 expression (intensity or percentage) and clinical outcome, including disease-free survival and overall survival, based on Kaplan-Meier survival analysis in high-grade serous carcinomas (data not shown). 

**Figure 4:**
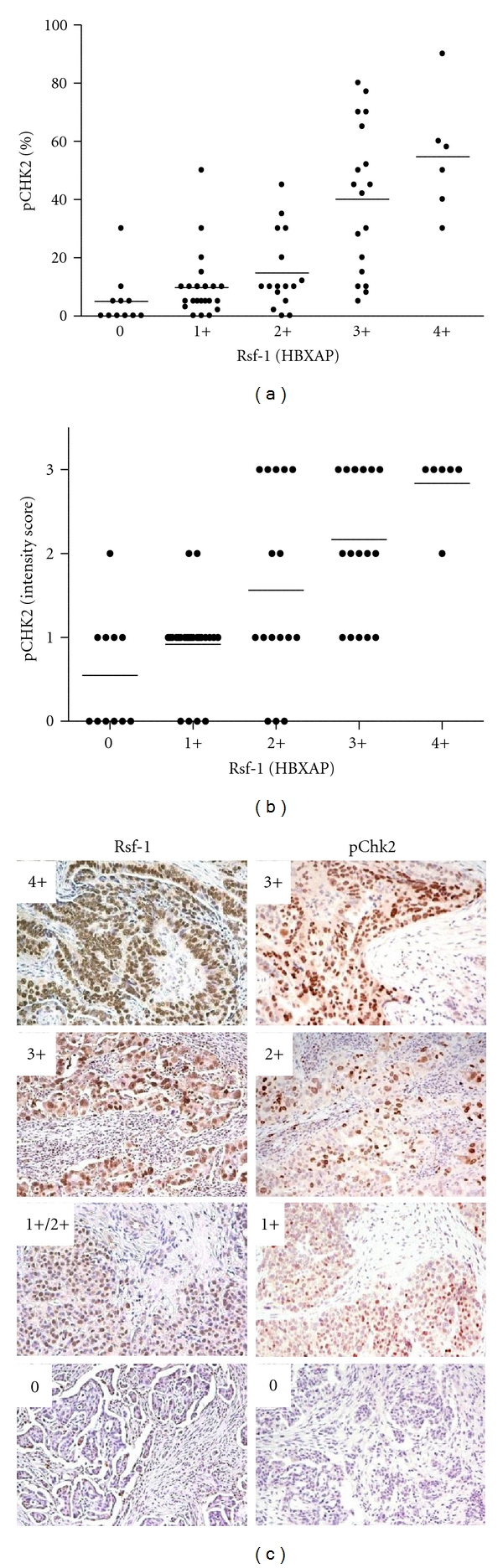
Correlation of the expression levels of pChk2 and Rsf-1 (HBXAP). (a) Scatter plots showing the distribution of pChk2 (percentage) and Rsf-1 (HBXAP) (immunostaining intensity) in 75 high-grade serous carcinomas. The correlation between the percentage of pChk2 positive cells and Rsf-1 immunostaining intensity was significant (*P* < 0.0001). Horizontal bars: means. (b) Scatter plots showing the distribution of pChk2 intensity scores and Rsf-1 (HBXAP) intensity scores in the same set of high-grade serous carcinomas. The correlation between immunostaining intensity of pChk2 and Rsf-1 was significant (*P* < 0.0001). Horizontal bars: means. (c) Four representative cases of high-grade serous carcinomas showing different staining patterns of pChk2 and Rsf-1. The intensity scores are shown in the left upper corner of each panel.

We then tested the correlation between the expression levels of pChk2 and Rsf-1 (HBXAP), because we have recently shown that amplification and overexpression of Rsf-1 (HBXAP) contribute to chromosomal instability by inducing DNA strand breaks [20]. Rsf-1 immunostaining results were available in 75 primary high-grade serous carcinomas from our previous study [17], enabling correlation with pChk2 data. Because Rsf-1 immunoreactivity is usually homogenous, we used intensity scores for Rsf-1 expression as previously described [17]. Rsf-1 immunostaining intensity significantly correlated with both percentage (*P* < 0.0001) and intensity (*P* < 0.0001) of pChk2 (Figures 4(a) and 4(b)). Representative immunostained tumor sections of pChk2 and Rsf-1 from the same tissues are shown in Figure 4(c). 

## 4. Discussion

It has been well established that DNA damage in cancer cells and associated DNA damage response pathway activation play an important role in chromosomal instability and tumor development [21–24]. The results from this study underscore the fundamental molecular differences between high-grade and low-grade serous carcinomas in terms of DDR activation. We have previously proposed that high-grade and low-grade serous carcinomas develop along distinct pathways [3]. Low-grade serous carcinoma develops from the precursor lesion, serous borderline tumor, tends to present at early stages, and is slow growing. In contrast, high-grade serous carcinoma, which has been generally referred to as ovarian cancer, behaves in a highly aggressive fashion, almost always present at advanced stages, and is associated with a dismal clinical outcome. They are typically not associated with borderline tumors, and in fact, a growing body of evidence has supported the view that many high-grade serous carcinomas develop from serous tubal intraepithelial carcinomas and involve the ovary secondarily [25–29]. At the molecular genetic level, low-grade serous carcinomas are characterized by frequent somatic sequence mutations in genes that are involved in signal transduction including *KRAS*, *BRAF*, *ERBB2,* and *PIK3CA* [1, 30–33]. In contrast, mutations in those genes are rarely detected in high-grade serous carcinomas; however, almost all high-grade serous carcinomas harbor *TP53* mutations. In addition to unique sequence mutations, high-grade serous carcinomas and low-grade serous carcinomas (the prototype of type I tumors) have distinct transcriptome and methylation profiles [3, 34, 35]. 

The higher pChk2 expression levels in high-grade serous carcinomas suggest that DDR is prominent in high-grade serous carcinomas as compared to low-grade serous carcinomas. This is likely due to frequent DNA damage in high-grade serous carcinomas. The pronounced DDR in high-grade serous carcinomas may be related to DNA replication stress due to activation of oncogenes and telomere shortening among several others [36, 37]. Furthermore, high-grade serous carcinomas usually have higher proliferative activity than low-grade serous carcinomas, precipitating the effects of DNA replication stress. On the other hand, tumor microenvironmental changes such as oxidative stress, hypoxia, and the presence of cytotoxic agents may potentiate DNA damage. Furthermore, recent evidence has suggested that excessive remodeling of chromosomal structures can be related to DNA strand breaks followed by DDR. In fact, we have recently shown that overexpression of a chromatin remodeling gene, Rsf-1 (HBXAP), leads to DNA double-strand breaks and DDR, resulting in p53-depdenent cell cycle arrest and apoptosis in *TP53* wild type, nontransformed cells [20]. DNA damage goes unchecked, and cells continue proliferating in the presence of DNA strand breaks if *TP53* is mutated, as what occurs in the great majority of high-grade serous carcinomas. Thus, the result from the current immunohistochemistry study further supports the biological link between Rsf-1 upregulation and DDR in ovarian cancer tissues either by aberrant chromatin remodeling or by oncogene/replicative stress. As *TP53* mutation represents one of the very early event in the development of high-grade serous carcinoma, it is currently not clear if DNA damage occurs before or after *TP53* mutations, although it has been suggested that activation of Chk2 and other DDR members may precede p53 inactivation in human epithelial tumors [22, 38].

It has been demonstrated that high-grade serous carcinoma, as compared to low-grade serous carcinoma, exhibits a higher level of chromosomal instability as reflected by abnormal mitosis, DNA copy number alterations, and karyotypic abnormalities [39, 40]. Thus, the increased pChk2 immunoreactivity in high-grade serous carcinoma raises a possibility that in high-grade serous carcinoma cells, DDR may not be fully capable of repairing DNA damage and maintaining genomic integrity, as the DNA damage may overwhelm the DNA repair capacity. As a result, clonal selection favors tumor cells harboring sustained DNA damage at a level that allows cells to survive but at the same time causes chromosomal instability, serving as the driving force in the evolution of highly aggressive tumor cell species. The finding of pChk2 expression in the majority of high-grade serous carcinomas implies that it may serve as a therapeutic target because inhibiting Chk2 may reduce DNA repair and increase genomic instability to a level that is not compatible with cellular survival and ultimately leads to tumor cell death. Indeed, it has been recently reported that a newly developed Chk2 small compound inhibitor could potentiate the cytotoxicity effect of PARP inhibitors, a finding supporting this view [41]. 

The majority of low-grade serous carcinomas do not contain prominent pChk2 immunoreactivity. Although our favored view is that less DNA damage is present, and, thus, attenuated DDR occurs in those tumors, other interpretations should also be pointed out. For example, the lack of pChk2 immunoreactivity might be a consequence of inactivation of upstream components in the DDR pathway [36]. As a result, ineffective DDR is present despite DNA damage in tumor cells. However, this scenario is less likely because unchecked and unrepaired DNA damage is generally detrimental to cells and is incompatible with sustained survival and proliferation in tumor cells. 

In summary, using pChk2 immunoreactivity as a surrogate marker for DDR, we found that high level DDR was detected more frequently in high-grade serous carcinomas than in low-grade serous carcinoma. This finding provides further support to the view that both tumors are molecularly distinct and develop along different molecular pathways. The significant correlation of expression between pChk2 and Rsf-1 (HBXAP) suggests that excessive chromatin remodeling activity as a result of upregulation of Rsf-1 (HBXAP) is associated with DDR in high-grade serous carcinoma tissues, a result supporting our previous observations that Rsf-1 upregulation contributes to DNA strand breaks and subsequent DDR. Future studies should aim at deciphering the mechanisms responsible for prominent DNA damage in high-grade serous carcinomas as compared to low-grade serous carcinomas and perhaps other type I tumors. Although our current study did not demonstrate an association of pChk2 expression levels with overall survival and disease-free survival, future clinical studies should be conducted to assess the biological significance of pChk2 in ovarian cancer patients. 

## Figures and Tables

**Table 1:** Immunostaining intensity of pChk2 in ovarian serous tumors, normal ovaries, and fallopian tubes.

Staining intensity	Low-grade	High-grade	High-grade	SBT	Serous cystadenoma	Normal ovaries and FTE
	SC	SC (primary)	SC (recurrent)			
0	31 (73.8%)	47 (29.6%)	28 (30.7%)	16 (100%)	10 (100%)	5 (100%)
1+	5 (11.9%)	37 (23.3%)	15 (16.5%)	0	0	0
2+	3 (7.1%)	39 (24.5%)	18 (19.8%)	0	0	0
3+	3 (7.1%)	36 (22.6%)	30 (33%)	0	0	0

SC: serous carcinoma; SBT: serous borderline tumor; FTE: fallopian tube epithelium.

## Abbreviations

Ch: Chromosome
Chk2: Check point kinase 2
DDR: DNA damage response.
